# Anemia and high hematocrit are associated with in-hospital mortality in emergency department patients with suspected infection

**DOI:** 10.1186/cc13298

**Published:** 2014-03-17

**Authors:** M Jessen, S Skibsted, N Shapiro

**Affiliations:** 1 Aarhus University Hospital, Aarhus, Denmark; 2 Beth Israel Deaconess Medical Center, Boston, MA, USA

## Introduction

Anemia and its association with mortality among emergency department (ED) patients with suspected infection has to our knowledge never been investigated. We hypothesize that anemia as well as high hematocrit increases the risk of in-hospital death among these patients.

## Methods

A prospective observational study of adult ED patients with suspected infection presenting to an urban, academic medical center ED at Beth Israel Deaconess Medical Center, Boston, MA, USA. Inclusion criterion was clinically suspected infection at ED presentation. Patients were enrolled over a 1-year period. Laboratory and clinical data were collected at enrollment. Primary outcome was in-hospital mortality. Logistic regression was performed determining the independent mortality odds adjusting for confounders.

## Results

A total of 4,952 patients were enrolled with an in-hospital mortality rate of 4% and a mean age of 58 ± 21 years. In total, 4,683 (95%) patients had their hematocrit measured: 3,857 (82%) patients had a normal hematocrit (30 to 44%), 413 (8.8%) patients had 25 to 29% hematocrit, and 66 (1.4%) patients ≤24% hematocrit. A total of 248 (5.3%) had a high hematocrit (≥45%). After adjusting for age, present or prior cancer, diabetes I and II, end-stage renal disease, AIDS and liver disease, anemia remained an independent predictor of in-hospital mortality with odds ratios of respectively 1.7 and 2.5 with worsening anemia as well as high hematocrit, with odds ratio 2.3 (Table 1). The AUC for the model was 0.78.

**Table 1:** Effect of severity of anemia or high hematocrit on in-hospital mortality

Hematocrit	Mortality OR	*P *value
≥45%	2.3(1.3 to 4.1)	0.007
30 to 44%, ref	1.0	
25 to 29%	1.7(1.1 to 2.6)	0.019
< 20%	2.5(1.1 to 5.8)	0.032

## Conclusion

Anemia and elevated hematocrit seem to be associated with in-hospital mortality among patients with suspected infection. Treatment of high or low hematocrit as a part of the ED resuscitation could be a subject for further investigation.

